# Canalization of genetic and pharmacological perturbations in developing primary neuronal activity patterns

**DOI:** 10.1016/j.neuropharm.2015.07.027

**Published:** 2016-01

**Authors:** Paul Charlesworth, Andrew Morton, Stephen J. Eglen, Noboru H. Komiyama, Seth G.N. Grant

**Affiliations:** a Genes to Cognition Programme, Wellcome Trust Sanger Institute, Genome Campus, Hinxton, Cambridgeshire, CB10 1SA, UK; b Cambridge Computational Biology Institute, Department of Applied Mathematics and Theoretical Physics, University of Cambridge, Cambridge, CB3 0WA, UK

**Keywords:** Synapse, Neuron, Network, Mutation, Canalization

## Abstract

The function of the nervous system depends on the integrity of synapses and the patterning of electrical activity in brain circuits. The rapid advances in genome sequencing reveal a large number of mutations disrupting synaptic proteins, which potentially result in diseases known as synaptopathies. However, it is also evident that every normal individual carries hundreds of potentially damaging mutations. Although genetic studies in several organisms show that mutations can be masked during development by a process known as canalization, it is unknown if this occurs in the development of the electrical activity in the brain. Using longitudinal recordings of primary cultured neurons on multi-electrode arrays from mice carrying knockout mutations we report evidence of canalization in development of spontaneous activity patterns. Phenotypes in the activity patterns in young cultures from mice lacking the Gria1 subunit of the AMPA receptor were ameliorated as cultures matured. Similarly, the effects of chronic pharmacological NMDA receptor blockade diminished as cultures matured. Moreover, disturbances in activity patterns by simultaneous disruption of Gria1 and NMDA receptors were also canalized by three weeks in culture. Additional mutations and genetic variations also appeared to be canalized to varying degrees. These findings indicate that neuronal network canalization is a form of nervous system plasticity that provides resilience to developmental disruption.

This article is part of the Special Issue entitled ‘Synaptopathy – from Biology to Therapy’.

## Highlights

• Development of network activity in cultures with synaptic mutations was recorded.
• Synchronous burst firing with theta periodicity was observed as cultures matured.
• *Gria1* deletion and chronic NMDA-R blockade disrupted network activity patterns.
• *Dlg2* knockout disrupted network activity, other synaptic genes had minimal effects.
• Network activity phenotypes early in development were canalized as cultures matured.

## 1 Introduction

Although large-scale human genome sequencing has identified hundreds of mutations causing brain disorders, all normal human individuals express a large number of highly damaging deleterious variants and disease-relevant mutations (Sulem et al., 2015, Xue et al., 2012). This raises the intriguing question: how does the brain maintain normal function in the presence of these mutations? Almost 70 years ago, Conrad Waddington recognised that mutations were masked during development and introduced the concept of ‘canalization’ to describe this inherent robustness (Waddington, 1942). Waddington developed the concept of canalization to describe the means by which developmental systems are channelled along a pathway or trajectory to their mature form. Deviations from this trajectory, caused by genetic or environmental perturbations, are diminished or canalized into developmental channels that lead to the final developed organism. Canalization not only allows organisms to develop and function normally in the presence of mutations but also hides genetic diversity in a population of phenotypically similar organisms, until it is unmasked by conditions of environmental stress and generates phenotypic diversity (Siegal and Bergman, 2006). Canalization is a self-organizing property of complex systems that is fundamentally different to homeostasis. Homeostasis maintains the stability of systems (using negative feedback) and canalization channels the system to a future and distinct set point. Although canalization has been recently studied in bacteria (Maisnier-Patin et al., 2005), yeast (Deutscher et al., 2006, Wagner, 2000) and *Drosophila* (Rutherford and Lindquist, 1998), little is known about its role in neural systems of vertebrates.

Cultured rodent primary neurons have been used extensively to study homeostatic mechanisms regulating neuronal excitability and firing patterns. It has been shown that networks of neurons stabilize their firing patterns in the face of environmental changes (Slomowitz et al., 2015). In hippocampus and cortex primary neuronal cultures from rodents, network synchrony can be perturbed by pharmacological manipulations, but typically returns over the course of hours (Kaufman et al., 2014).

To our knowledge there has been no direct experimental evidence showing canalization of mutation in the development of bursting and firing patterns in neural circuits. In the course of developing an *in vitro* assay for the purposes of studying the impact of disease-relevant mutations on neuronal circuits (MacLaren et al., 2011) we unexpectedly observed evidence for canalization. We monitored the activity of developing neural circuits, from birth to 4 weeks of age, in a tissue culture chamber where a 59 electrode array (MEA, Multi-electrode array) was overlaid with primary cultures of mouse hippocampal neurons. Using this approach, we have previously correlated longitudinal recordings of firing patterns and synchronization in neuronal networks with underlying changes in gene expression (Valor et al., 2007) and characterized differences in the network activity profiles of hippocampal versus cortical neurons (Charlesworth et al., 2015). Here we report experiments with mutations in these assays, using primary cultures from mice carrying knockout mutations in a glutamate receptor subunit, and multiple post-synaptic scaffolds and signalling proteins.

## 2 Materials and methods

### 2.1 Preparation of multi-electrode arrays

On the day of plating, planar multi-electrode arrays (59 titanium nitride electrodes, 30 μm-diameter, 200 μm-spacing, internal reference electrode; MultiChannel Systems, Fig. S1) were sterilized in a plasma cleaner (Diener Electronic). The central-most portion of the culture compartment was treated with an 8 μl drop of poly-d-lysine (Sigma) (0.5 mg/ml), washed with 8 μl sterile water, then coated with a 4 μl drop of ice-cold 1 mg/ml laminin (Invitrogen). 30 μl of full Neurobasal medium was dispensed round the perimeter of the culture compartment of the MEA prior to the laminin coating step. MEAs were fitted with a sterile, gas-permeable/water vapour-impermeable lid (Potter and DeMarse, 2001) and placed in an incubator (37 °C; 5%CO_2_/95% air; humidified) until required for plating.

### 2.2 Primary neuronal cultures

Primary cultures of dissociated hippocampal neurons were prepared from embryonic day (E) 17–18 mice. Pregnant mice from timed matings were killed by cervical dislocation and embryos were removed and decapitated before dissecting hippocampi from embryonic brains, keeping tissues submerged in ice-cold Dulbecco's phosphate buffered saline (DPBS) (Invitrogen) containing 1% v/v penicillin/streptomycin solution (Invitrogen). For wild-type or null mutants, hippocampi from multiple embryonic brains were pooled. Following incubation at 37 °C in 10 units/ml papain (Worthington) for 22 min, hippocampal tissue was disaggregated by reverse pipetting in pre-warmed suspension medium (Dulbecco's Modified Eagle's Medium/F:12 (1:1) containing 10% v/v foetal bovine serum and 1% v/v penicillin/streptomycin solution). This cell suspension was centrifuged at 400 × *g* for 3.5 min, the pellet re-suspended in fresh suspension medium, and centrifuged a second time at 400 × *g* for 3.5 min. The final pellet was resuspended (100 μl/pair hippocampi) in pre-warmed full Neurobasal medium (Neurobasal, B27 supplement, 500 μM l-glutamine, 1% v/v penicillin/streptomycin solution). Cell yield was counted using a haemocytometer before seeding 2 × 10^5^ cells (typically equating to around 30 μl of cell suspension) in the centre of multi-electrode arrays (prepared as described above) containing 600 μl full Neurobasal medium. Zero-evaporation lids were refitted and the MEAs housed in tissue culture incubators maintained humidified at 37 °C and 5% CO_2_/95% air. At 3–4 DIV, cultures were fed by replacing 200 μl medium with pre-warmed fresh full Neurobasal medium. Cultures were subsequently fed using the same method after each recording, equating to a one third medium change twice per week. Where cultures were chronically treated with APV (50 μM), this was added when cultures were fed after recording at 7 DIV, and then maintained at this concentration with subsequent feeds.

24 h post-plating, MEAs were placed on an inverted microscope with heated stage (Axiovert 200; Zeiss) and photographed through a 32× phase objective at 5 different fields of view (Fig. S1b). To confirm similar numbers of adherent cells between preparations, automated cell counting of these images was performed using a pipeline designed in CellProfiler (Carpenter et al., 2006). After completion of time-courses, cells were trypsinated, pelleted as described above in DMEM/F-12/FBS and resuspended in Wizard SV lysis buffer (Promega) for confirmatory genotyping by PCR.

### 2.3 Mouse lines

All procedures were performed in accordance with the United Kingdom Animals (Scientific Procedures) Act 1986. The mouse lines used in this study were as follows:

Wild type

C57BL/6-*Tyr*^*c*-*Brd*^ (C57; albino C57BL/6), 22 cultures, 76 MEA platings

129S5/SvEvBrd (129S5), 13 cultures, 53 MEA platings

Mutant lines (homozygous null breedings)

*Gria1*, (C57 background (Zamanillo et al., 1999)), 15 cultures, 68 MEA platings

*Arhgap32*, (C57 background), 3 cultures, 26 MEA platings

*Dlg2*, (C57 background (McGee et al., 2001)), 10 cultures, 71 MEA platings

*Gnb1*, (129 background), 3 cultures, 26 MEA platings

*Dlg4*, (129 background (Migaud et al., 1998)), 10 cultures, 40 MEA platings

*Dlg3*, (129 background (Cuthbert et al., 2007)), 9 cultures, 52 MEA platings

*Sipa1l1*, (129 background), 5 cultures, 41 MEA platings

We confirmed that the divergent genetic backgrounds of the two wild-type strains used in this study exerted no detectable influence on the electrophysiological parameters measured (see Fig. S6).

### 2.4 Multi-electrode array recording

Multi-electrode arrays and all data acquisition hardware and software were from MultiChannel Systems (Reutlingen, Germany). Pairs of MEAs were interfaced with duplex 60 channel amplifiers and 15 min recordings of spontaneous action potentials were made twice per week during the four weeks following plating. MEAs were heated and kept under a light flow of 5% CO2/95% air during recordings. Signals were digitized with a 128-channel analogue/digital converter card at a rate of 25 kHz and filtered (100 Hz High pass) to remove low frequency events and baseline fluctuations. Action potentials were detected by crossing of threshold set to a fixed level of −20 μV, which typically approximated to 6–8 standard deviations from the baseline noise level. Record samples (1 ms pre- and 2 ms post-crossing of threshold) confirmed the characteristic action potential waveform. Application of tetrodotoxin (TTX, 1 μM) totally abolished spiking activity, confirming the absence of false positive event detection using these methods. Network activity was also silenced by acute co-application of APV (50 μM) and DNQX (20 μM) confirming it was predominantly mediated by glutamatergic neurotransmission (see Fig. S4). Furthermore, *Gria1*^−/−^ cultures showed a diminished capacity to generate “network spikes” (see below and Fig. S3) suggesting a requirement for fast glutamatergic neurotransmission for network-wide communication. Spikes were not sorted to distinguish signals generated by individual neurons, and as such represent multiunit activity (see Fig. S5).

### 2.5 Data analysis

#### 2.5.1 Spike train extraction and burst detection

Action potential timestamps were extracted using batch scripts written for NeuroExplorer (Nex Technologies, Littleton, MA) and analysed using custom-written software developed in the R statistical programming environment (R Core Team, 2014) to compute parameters that quantitatively describe network activity. Full details of these analysis methods, including scripts accompany an earlier paper (Charlesworth et al., 2015). All spike trains analysed in this paper are freely available from the CARMEN portal (https://portal.carmen.org.uk; see Supplementary Information for access details).

A burst-detection algorithm similar to the “max interval method” used in NeuroExplorer was implemented to classify trains of action potentials with these characteristics as bursts. This method parses a spike train into bursts based on various thresholds for the interspike interval (ISI) between spikes starting and ending a burst, plus thresholds for deciding when to merge bursts. The principal parameters used in this analysis were: Minimum interspike interval between bursts = 800 ms; min spikes/burst = 6; min burst duration = 50 ms.

For each 15 min recording, the following network parameters were initially calculated:

#### 2.5.2 Total spikes

The sum of the total number of spikes detected by all electrodes in each recording.

#### 2.5.3 Network size

The total number of electrodes recording bursting activity at a rate greater than 1 burst per minute.

#### 2.5.4 Percentage spikes in bursts

The percentage of spikes organized within bursts.

#### 2.5.5 Burst pattern

The coefficient of variation of the inter-burst interval (CV IBI), which provides a measure of the temporal regularity of activity. The intervals between bursts of spikes are averaged across the whole recording for each electrode. From this list of values, a coefficient of variation is calculated, with higher values reflecting a lack of temporal structure to activity and values closer to zero indicating temporal organization.

#### 2.5.6 Burst duration

The average duration of the bursts detected (in seconds), as classified by the burst-detection algorithm described above.

#### 2.5.7 Burst rate

Represented per minute, the rate at which bursts occur averaged across all active electrodes.

#### 2.5.8 Correlation index

Correlation index measures the coincidence of spikes in each electrode pair (maximum 1711) of the array, based on the method described in Wong et al. (1993).

#### 2.5.9 Network spikes

For *Gria1*^−/−^ cultures we also performed an exploratory analysis of network-wide events: “Network spikes” essentially average the spiking activity across all active channels into one merged channel (Eytan and Marom, 2006). Spike times across all channels are binned into small intervals (3 ms in the present study) and then the population trace is examined to find peaks of activity when the number of active electrodes exceeds a threshold, set in this study to 10 electrodes.

### 2.6 Statistics

Confidence intervals (2.5%, 97.5%) for the network parameters were calculated by bootstrap resampling (with replacement) using a script written in R. P values were then calculated by T test to the mean of the bootstrap distribution.

To weight differences observed in proportion to their statistical significance, at each time-point, for the network parameters described above (except network spikes), phenotypic effect size (PES) was calculated as:

$$\text{PES}=\frac{\text{Wild}-\text{type}\;\text{lower}\;\text{confidence}\;\text{interval}–\text{Mutant}\;\text{upper}\;\text{confidence}\;\text{interval}}{\text{Wild}-\text{type}\;\text{median}}$$

Or for changes of opposite directionality:

$$\text{PES}=\frac{\text{Mutant}\;\text{lower}\;\text{confidence}\;\text{interval}–\text{Wild}-\text{type}\;\text{upper}\;\text{confidence}\;\text{interval}}{\text{Wild}-\text{type}\;\text{median}}$$

In cases where the confidence intervals of wild-type and mutant overlapped, PES gave a negative value, which was treated as zero for the purposes of calculating the total phenotypic effect size (PES^*total*^):

$${\text{PES}}^{\text{total}}=\Sigma \;\text{PES}\;\text{for}\;\text{all}\;\text{parameters}$$

## 3 Results and discussion

**Fig. 1:**
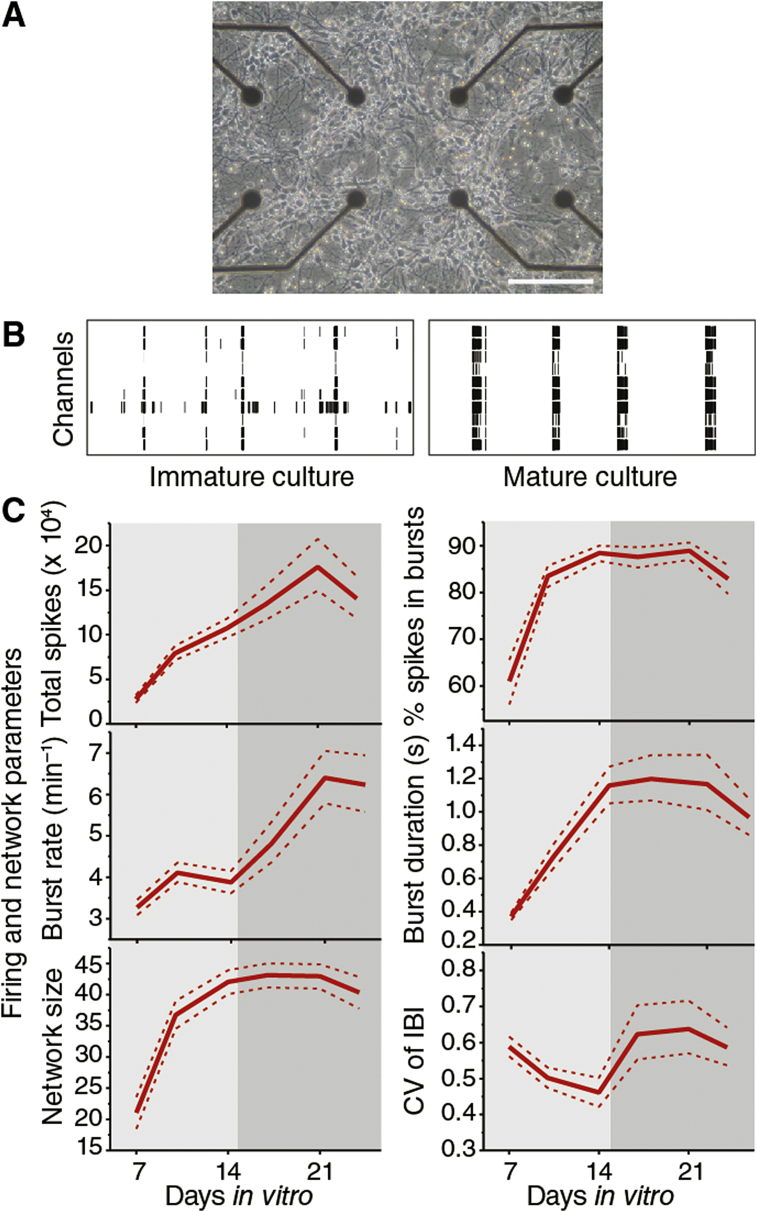
Overview of experimental system and example results. **A**. Example phase contrast micrograph of primary dissociated embryonic hippocampal neurons on a multi-electrode array. Scale bar = 0.2 mm. **B**. Example raster plots showing 60 s of activity from the first 10 channels of recordings made at 7 days *in vitro* (DIV) (immature culture) and 21 DIV (mature culture). **C**. Developmental trajectories (between 7 DIV and 24 DIV) of the core firing and network parameters recorded longitudinally in this study, for the pooled WT dataset. Solid red lines denote the bootstrap mean and dashed red lines show ±2.5% confidence intervals (see Materials and Methods). Total spikes reports the sum of spikes recorded on all MEA channels throughout 15 min recording epoch. Burst rate is the mean number of bursts detected per minute (see Materials and Methods for details of the burst detection algorithm used). Network size is the number of electrodes detecting >1 burst per minute. % spikes in bursts is the percentage of individual spikes that occurred within bursts and burst duration is the mean duration of bursts in seconds. CV of IBI is the coefficient of variation of the inter burst interval. (For interpretation of the references to colour in this figure legend, the reader is referred to the web version of this article.)

To measure the impact of mutations on developing network activity, longitudinal recordings were made from multi-electrode arrays (Figs. 1A and S1). Primary cultures of dissociated mouse embryonic hippocampal neurons were plated on arrays and recordings were made of spontaneous network activity from each culture twice per week for 3–4 weeks. Several network parameters were derived from the resultant spike patterns and used to construct developmental time courses of network activity levels, burst patterns and network synchrony. Phenotypes of wild-type (WT) networks were compared with those derived from mice carrying mutations in neurotransmitter receptor subunits, scaffolding proteins and other signalling molecules. The phenotypic effect size (PES) was monitored for each mutation across development enabling us to determine the overall magnitude of the mutant phenotypes, and also whether these changed with age.

MEA recordings from hippocampal neurons in culture are initially electrically silent and over four weeks (representing the normal maturation time course *in vivo*) generate highly patterned spontaneous activity (Fig. 1B). A stereotypical developmental profile of two broad phases – growth and stabilization – is observed in the activity patterns quantified using six parameters that describe spiking, bursting and network synchrony (Fig. 1C). The transition between the growth and stabilization phase occurs around 14 days *in vitro* (DIV) (Fig. 1C). The mature, stabilized cultures show highly synchronized activity (Fig. 1B and C) including prominent theta bursting (Fig. S2). Of the six parameters, three (network size, spikes in bursts, burst pattern) showed greatest change during the growth phase and were stable thereafter, whereas total spikes and burst rate increased over 21 days to the mature stable level (Fig. 1C) in accordance with previous observations (Valor et al., 2007).

**Fig. 2:**
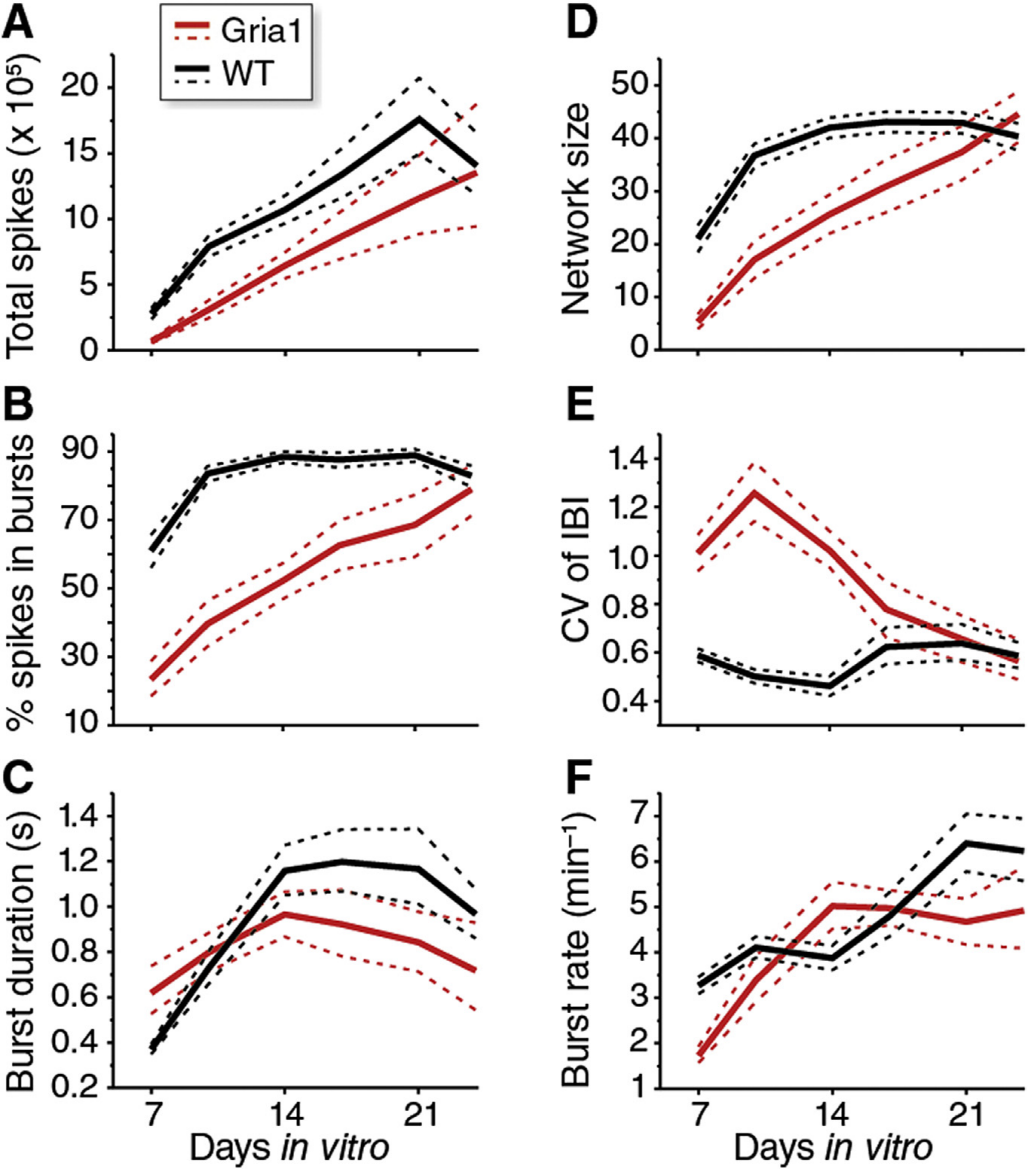
Comparison of developmental profiles of network activity parameters in *Gria1*^−/−^ and wild-type (WT) cultures. In all graphs, the solid lines denote mean values and dashed lines denote 97.5% confidence intervals of the mean assessed by resampling (see Materials and Methods). *Gria1*^−/−^ data are plotted with red lines and WT data with black lines. **A**. Total spikes recorded from all active electrodes during each 15 min recording. **B**. Percentage of spikes recorded that were contained within bursts. **C**. The mean duration, in seconds, of bursts detected. **D**. Network size, defined as the number of electrodes in each recording with a burst rate >1 min^−1^. **E**. CV of IBI, the coefficient of the interburst interval. **F**. Burst rate, the mean number of bursts per minute. (For interpretation of the references to colour in this figure legend, the reader is referred to the web version of this article.)

**Fig. 3:**
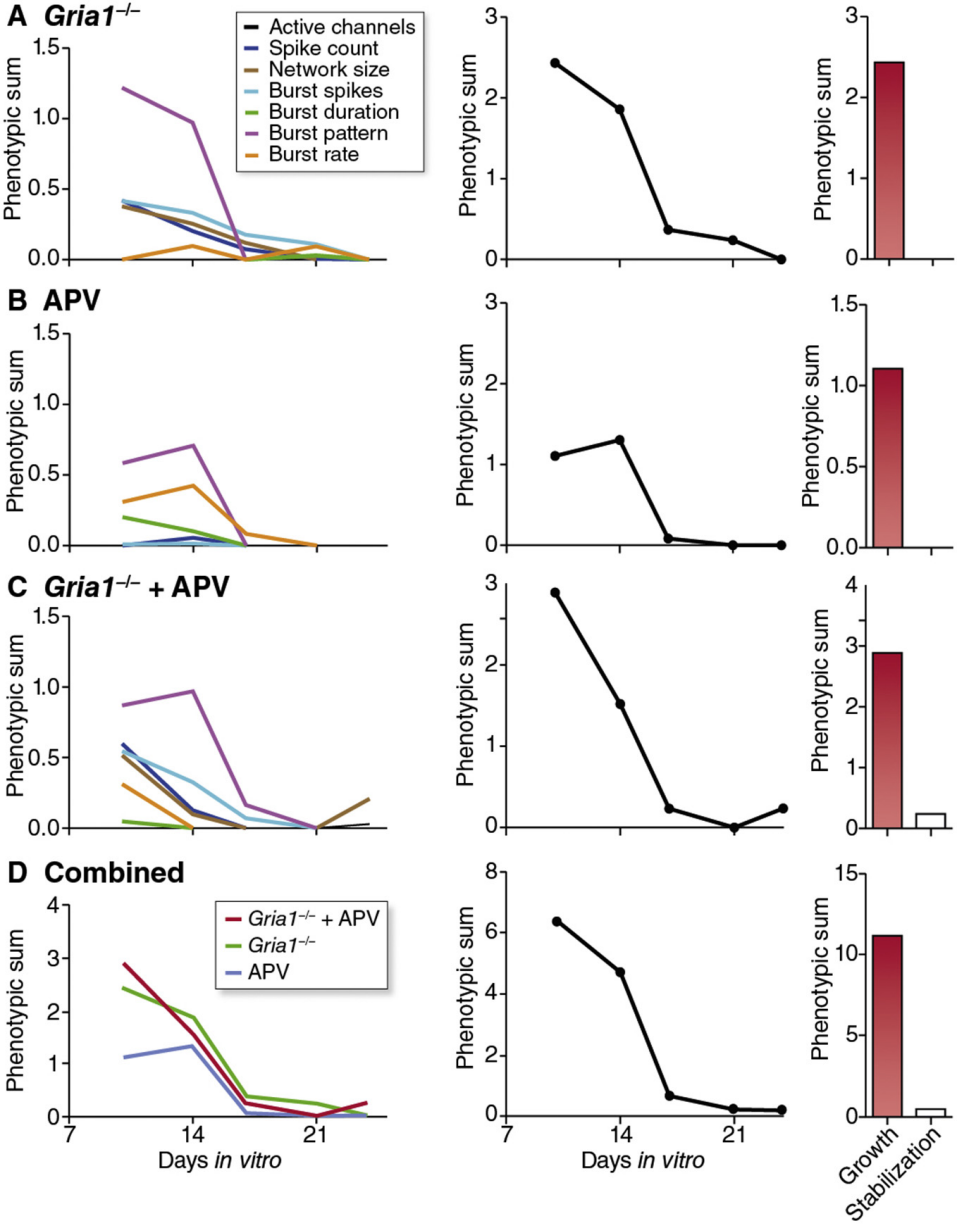
Disrupted network synchrony during development of Gria1^−/−^ cultures is mimicked by chronic NMDA receptor blockade and reduced at maturity by NMDA-receptor independent mechanisms. **A**. PES (phenotypic effect size) comparison between wild-type cultures and *Gria1*^−/−^ cultures (see Materials and Methods for calculation). Left panel shows PES for individual network parameters (see Materials and Methods for details of parameters). Middle panel is summed PES (PES^total^) for all parameters. In the right panel, bar height is the summed PES^total^ from growth (DIV 10 and 14 timepoints) and stabilization (DIV 21 and 24 timepoints) phases. **B**. Plots are as in A, showing PES for wild-type cultures maintained chronically in 50 μM D-APV. **C**. Plots are as in A, showing PES for *Gria1*^−/−^ cultures maintained in 50 μM D-APV throughout the experimental timecourse. **D**. Merged summary plot showing PES^total^ for all three perturbations.

Using this system we initially studied a homozygous knockout mutation in *Gria1* (*Gria1*^−/−^) (Zamanillo et al., 1999), which is a major subunit of the AMPA receptor (AMPA-R) and expected to have a robust phenotype since it plays a central role in synaptic transmission and plasticity (Huganir and Nicoll, 2013). Multiple network parameters were indeed disrupted by DIV10, most notably the percentage spikes in bursts (Fig. 2B), network size (Fig. 2D) and the CV of IBI (coefficient of the interburst interval) (Fig. 2E). Furthermore, a substantially-reduced incidence of network spikes (synchronous firing at >10 electrodes) was observed in *Gria1*^−/−^ cultures (Fig. S3). Overall spike activity was also reduced (Fig. 2A). The disruption to these parameters largely reflects the reduced tendency for network activity in *Gria1*^−/−^ cultures to be structured in bursts. Intriguingly however, as cultures matured, a number of these mutant phenotypes progressively disappeared. We quantified the differences between mutant and WT cultures by calculating a phenotypic effect size (PES) for each parameter and timepoint. PES of the four most disrupted parameters at DIV10-14 (spikes, network size, pattern, burst spikes) progressively returned to control values by DIV25 (Fig. 3A, left panel; Table S2). As a measure of overall phenotype effect size, we summed individual network parameters (PES^total^), which showed a reduction during the transition from the growth to stabilization phase (Fig. 3A, middle and right panels). Therefore, *Gria1*^−/−^ exhibited a strong neuronal network phenotype during the growth phase that was canalized during the stabilization phase.

Since the NMDA receptor (NMDA-R) is well known to control synaptic plasticity following changes in activity and regulates AMPA-Rs (Luscher and Malenka, 2012), we reasoned that the apparent canalization of the *Gria1*^−/−^ phenotype might be mediated by this receptor. We tested this hypothesis by adding an NMDA-R antagonist, D-(−)-2-Amino-5-phosphonopentanoic acid (APV) to developing cultures. First, we characterized the effect of chronic administration of APV on the development of WT activity patterns. By DIV10 (after 3d exposure to APV) we observed a marked impairment in burst pattern (CV IBI) and also increased asynchronous non-burst spike firing (Fig. 3B, Table S2), consistent with previous reports using APV on MEA cultures (Keefer et al., 2001). Surprisingly however, these pharmacologically-induced changes were also canalized as cultures matured (Corner et al., 2005) (Fig. 3B, middle and right panels). Further, when we applied APV to *Gria1*^−/−^ cultures, the phenotypic effect of combined perturbation of NMDA-R and AMPA-R signalling did not prevent canalization of the disruptions to network activity patterns observed early in the development of *Gria1*^−/−^ cultures (Fig. 3C and D).

**Fig. 4:**
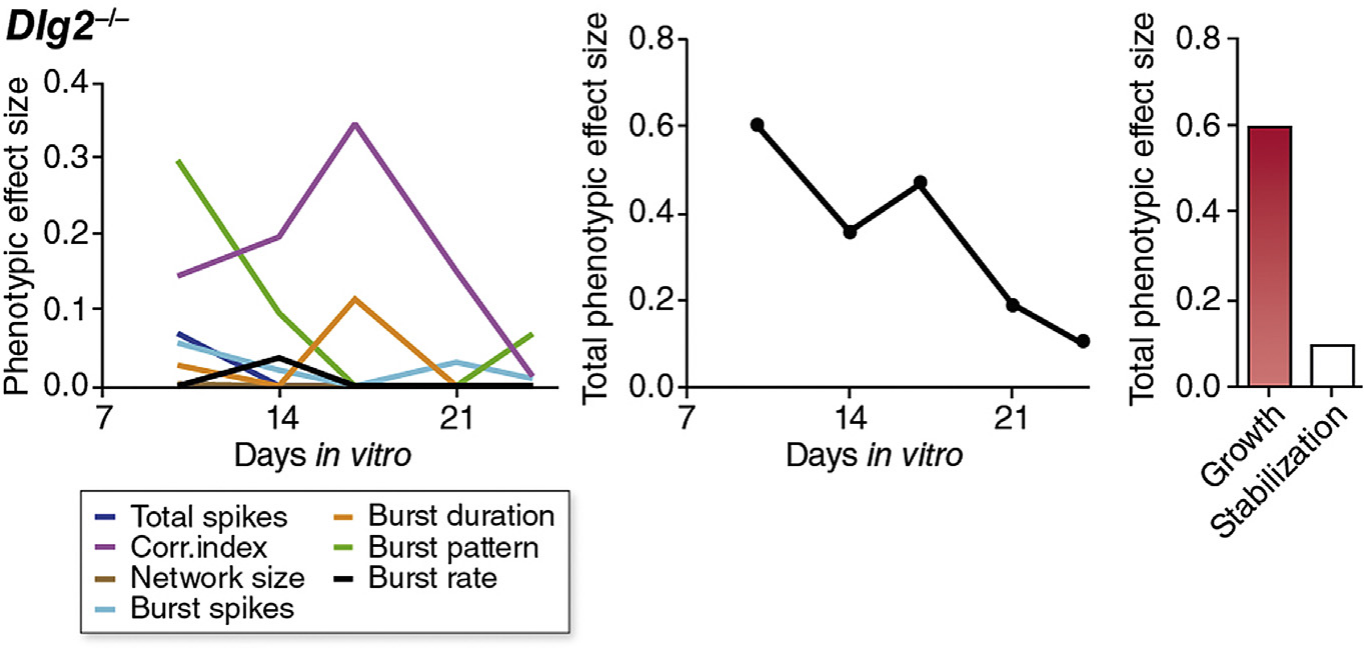
Developmental profile of the *Dlg2*^−/−^ phenotype. Left panel, phenotypic effect size of network parameters in *Dlg2*^−/−^ cultures from 10 DIV to 24 DIV. Middle panel is the summed phenotypic effect size (PES^total^) for all parameters. In the right panel, bar height is the summed PES^total^ from growth (DIV 10 and 14 timepoints) and stabilization (DIV 21 and 24 timepoints) phases.

These results suggest that developing neuronal networks may have developmental plasticity mechanisms that refine activity patterns and that this mechanism can overcome genetic and pharmacological perturbations. Moreover, our findings show these canalization mechanisms are not only independent of the principal glutamatergic synaptic plasticity mechanisms, but can overcome disruption in these key mechanisms. We next asked if canalization was observed for mutations in other types of genes by studying cultures from mice carrying null mutations in six genes (*Arhgap32*/GRIT, *Dlg2*/PSD93, *Dlg3*/SAP102, *Dlg4*/PSD95, *Gnb1*/GNB1, *Sipa1l1*/SIPA1) representing other classes of proteins (Tables S1 and S2). We found that the phenotypic effect of many of these mutations was small (*Arhgap32*/GRIT, *Dlg4*/PSD95), or negligible (*Dlg3*/SAP102, *Gnb1*/GNB1, *Sipa1l1*/SIPA1), probably due to compensatory gene expression (Table S2). An interesting exception was *Dlg2*/PSD93, which displayed significant perturbations in most network parameters during the growth phase, but, as with *Gria1* knockout and NMDA-R blockade, almost complete canalization of these phenotypes at maturity (Fig. 4 and Table S2).

Thus, those manipulations that had a major impact on network properties during growth, also revealed a very strong capacity for those deleterious effects to diminish with maturation.

Since the mice used in this study were on two different backgrounds it was important that we tested for potential network differences between these two strains of WT mice (C57BL/6J and 129S5). Interestingly, despite their widespread genomic differences (Keane et al., 2011), we found no significant differences between network activity patterns from the two strains throughout their development (Fig. S6).

**Fig. 5:**
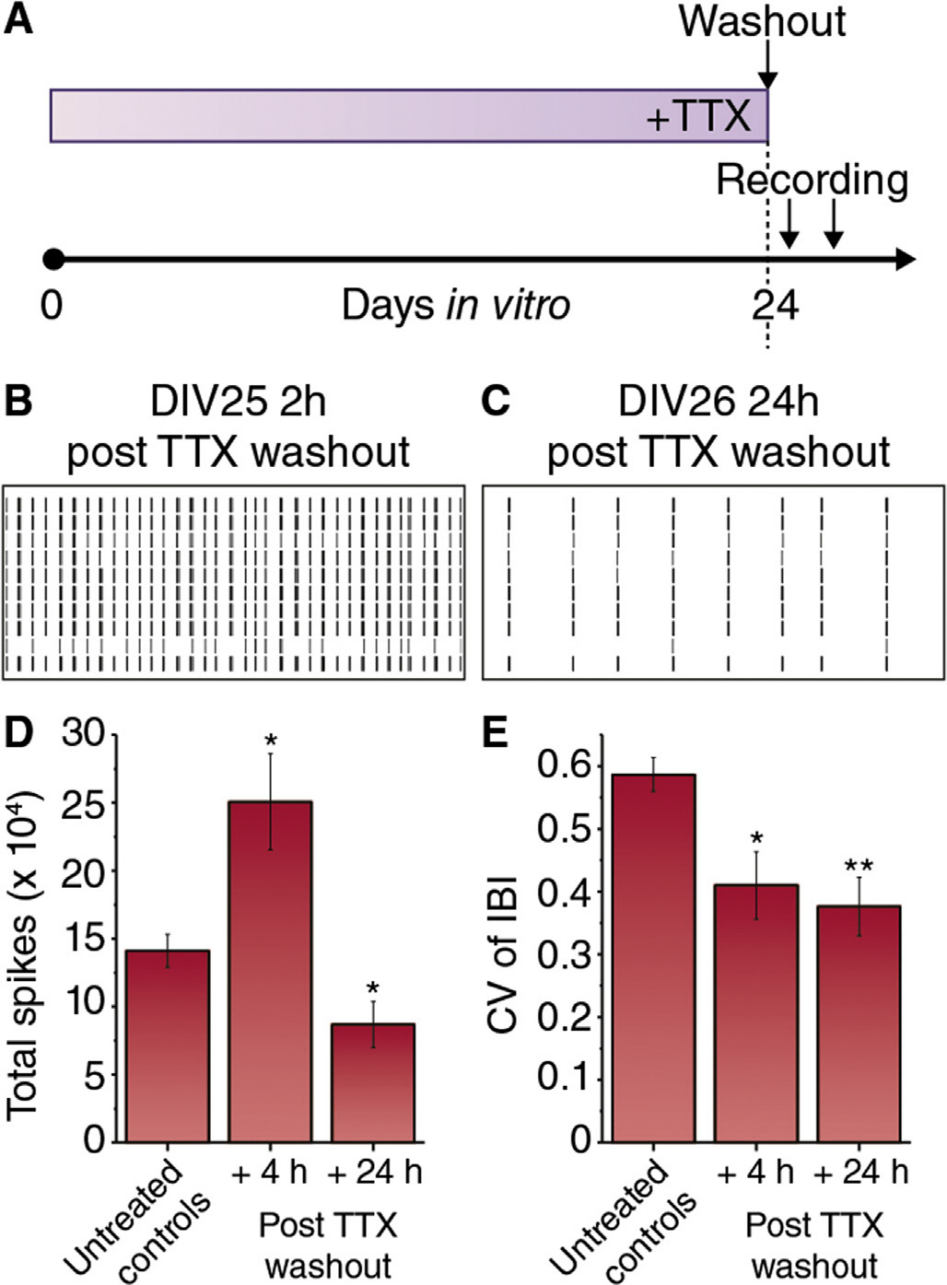
Maturation of network activity patterns does not require recurrent activity during development. **A**. Schematic of experimental design. Cultures were grown in culture medium supplemented with tetrodotoxin (TTX; 1 μM) until 24-25DIV, when TTX was washed out. Multielectrode array recordings were then made 2–4 h and 24 h post-washout of TTX. **B**. Raster plot showing 60 s of activity from first 10 electrodes from a representative recording made 2 h following TTX washout. **C**. As in B, from a recording made 24 h post TTX washout. **D**. Histogram of Total spikes following washout of TTX at DIV 25, compared to untreated controls (*P < 0.05 Students t-test with Welch's correction.) **E**. Burst pattern (CV of IBI) following washout of TTX at DIV 25, compared to untreated controls (*P < 0.05; **P < 0.01 Students t-test with Welch's correction).

An extensive body of literature shows neuronal activity shapes the maturation of the mammalian nervous system (see Cooke and Bear, 2014, Whitt et al., 2014 for recent reviews). Although many of these mechanisms require NMDA receptor function, which is dispensable for canalization, we reasoned that activity throughout development may be required for the networks to achieve their optimum pattern of activity. We therefore eliminated spiking activity during development by growing cultures in tetrodotoxin (TTX) from DIV0 (Fig. 5A). Such cultures remained silent until TTX was washed out at DIV24 (Fig. 5B and C). Within the first four hours following TTX washout, spike number was increased (P = 0.022; Fig. 5D) and firing patterns showed an unexpectedly enhanced regularity (Fig. 5E; P = 0.016). At 24 h after TTX washout, total spikes were significantly reduced relative to untreated cultures (Fig. 5D), but the temporal regularity of burst patterns remained higher than in untreated cultures (Fig. 5E). These observations, particularly in total spikes recorded, are likely reflective of anticipated homeostatic adaptations after chronic silencing of activity. However, they also suggest that the stable firing patterns observed in mature cultures can largely be established in the absence of prior recurrent network activity, implying that canalization of firing pattern perturbations is likely activity-independent. This principle could be tested directly by characterizing the activity-dependence of canalization of mutant network activity phenotypes, by combining the genetic and pharmacological approaches presented in this study.

Our study reveals a robust capacity for cultured neuronal networks to self-organize and develop synchronous ensemble burst firing in the face of perturbations. In line with developmental canalization, where the system organizes in the face of perturbation on a trajectory to its mature function, we observed resilience to different kinds of genetic and pharmacological perturbations. In contrast to homeostatic plasticity and synaptic plasticity, which require AMPA, NMDA receptors and activity, we found that blockade of Gria1 (a knockout mutation), NMDA receptor, spontaneous activity (pharmacological antagonists) as well as polygenic genetic variation did not prevent circuits from developing to stable firing patterns by four weeks. These findings indicate that canalization may operate as a form of plasticity in the nervous system.

As the pattern of spontaneous neuronal activity is thought to be important for the development of neuronal connectivity (Kirkby et al., 2013) canalization could help restore spontaneous activity to its normal pattern and thus limit any deleterious changes in the wiring of neural circuits. Furthermore, canalization could be important for cognition because it would facilitate optimal transmission of information; bursts are necessary to drive network activity and precision of spike timing is required for synaptic plasticity (Feldman Daniel, 2012). Neural network canalization could therefore stabilize and normalize behaviours by modifying the severity of phenotypes, keeping the complex brain functioning in the face of mutation and developmental damage. Both heritable and *de novo* mutations could be subject to canalization, which would diminish the phenotype during the early postnatal developmental period. Studies in *Drosophila* show that unmasking of canalized mutations exposes latent phenotypes (Rutherford and Lindquist, 1998) raising the speculation that canalization of disease-relevant genes during the development of the nervous system may mask a vulnerability that can be later exposed.

A number of different mechanisms have been proposed to play a role in canalization. Phenotypes caused by SNPs affecting protein structure are canalized in *Drosophila* by the HSP90 chaperone protein (Rutherford and Lindquist, 1998). Another general mechanism is gene duplication, where redundant paralogs in gene families obscure phenotypes (Koonin, 2005). A third mechanism is the robustness conferred by organization of molecular networks, such as gene regulatory networks (Bergman and Siegal, 2003) or protein interaction networks (Ideker and Sharan, 2008). Each of these mechanisms are present in the postsynaptic proteome of mammalian synapses (HSP90 (Collins et al., 2006), paralogs (Emes et al., 2008), molecular networks (Pocklington et al., 2006)) and upregulated prior to the onset of buffering in the stabilization phase in cultured neurons (Valor et al., 2007). Since mutations change the transcriptome of cells and hence their identity, it is possible that there are changes in the populations of cell types in the mixed cultures. Future studies manipulating these mechanisms at specific times in development will be necessary to determine their role in neuronal activity.
