# Supplementary material for: Canalization of genetic and pharmacological perturbations in developing primary neuronal activity patterns
Source: Neuropharmacology. 2016 Jan;100:47–55. doi: 10.1016/j.neuropharm.2015.07.027 (PMC4726661; doi:10.1016/j.neuropharm.2015.07.027)
Supplement: Supplementary file 1 [file mmc1.docx]

**SUPPLEMENTARY INFORMATION**

**Canalization of genetic and pharmacological perturbations in developing primary neuronal activity patterns**

Paul Charlesworth ^a,c,f^ , Andrew Morton ^a,d,f^, Stephen J. Eglen^b^, Noboru H. Komiyama^a,e^ & Seth G. N. Grant^a,e^

**Affiliations:**

1. Genes to Cognition Programme, Wellcome Trust Sanger Institute, Genome Campus, Hinxton, Cambridgeshire, CB10 1SA, UK.
2. Cambridge Computational Biology Institute, Department of Applied Mathematics and Theoretical Physics, University of Cambridge, Cambridge, CB3 0WA, UK.
3. Present address: Department of Physiology, Development and Neuroscience, Physiological Laboratory, University of Cambridge, Cambridge CB2 3EG, UK.
4. Present address: School of Physics and Astronomy, University of St Andrews, St Andrews, KY16 9SS, UK.
5. Present address: Centre for Clinical Brain Sciences, The University of Edinburgh, Edinburgh EH16 4SB, UK.
6. These authors contributed equally to this work

**Fig. S1.**

**Images of multi-electrode arrays.**

**a.** A photograph showing an example multi-electrode array as used in this study.

**b.** An example phase-contrast micrograph showing the central-most area of the multi-electrode array 1 day after cell plating. The spacing between electrodes is 200μm. These images are from the authors’ website (<http://www.genes2cognition.org>).


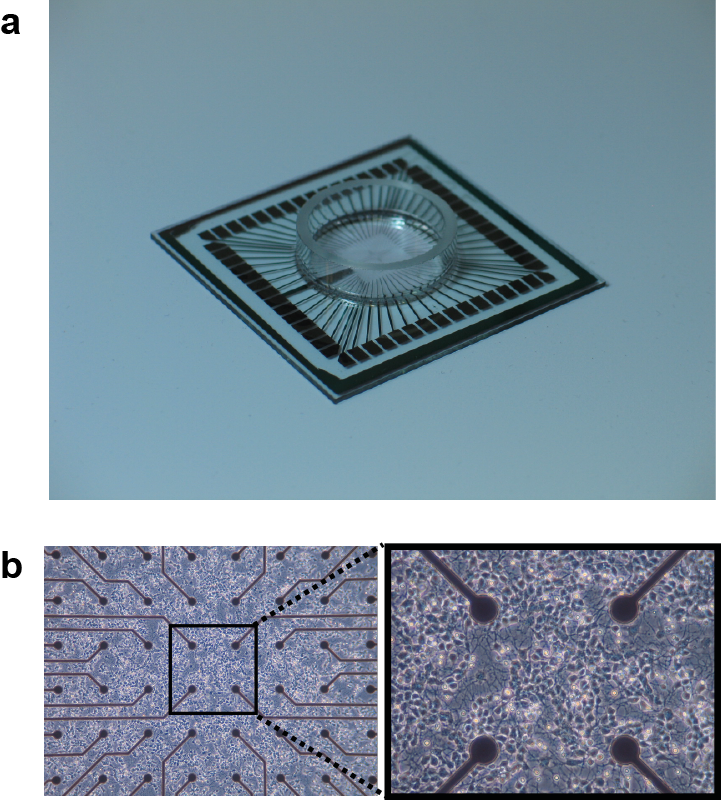


**Fig. S2.**

**Prominent burst-firing at theta periodicity in dissociated hippocampal neurons in culture.**

**a.** Recording from a wild type culture, aged 18 DIV. From a typical fifteen-minute recording, a three-second period of activity is shown. Bursts occur at a frequency in the range 6-10Hz, indicative of theta-bursting.

**b.** Density estimate of the interspike intervals (ISIs) from this recording (27706 intervals taken from 47 electrodes). The x-axis is plotted on a logarithmic scale, showing three prominent peaks. The leftmost peak, about 200 Hz (5 ms ISI), represents the average ISI within bursts. The middle peak at about 7Hz is the separation between bursts within periods of theta-bursting (approx. 150 ms ISI). The rightmost peak at around 0.04 Hz (ISI about 26 s) denotes the long delay between spontaneous bursts of activity.

**
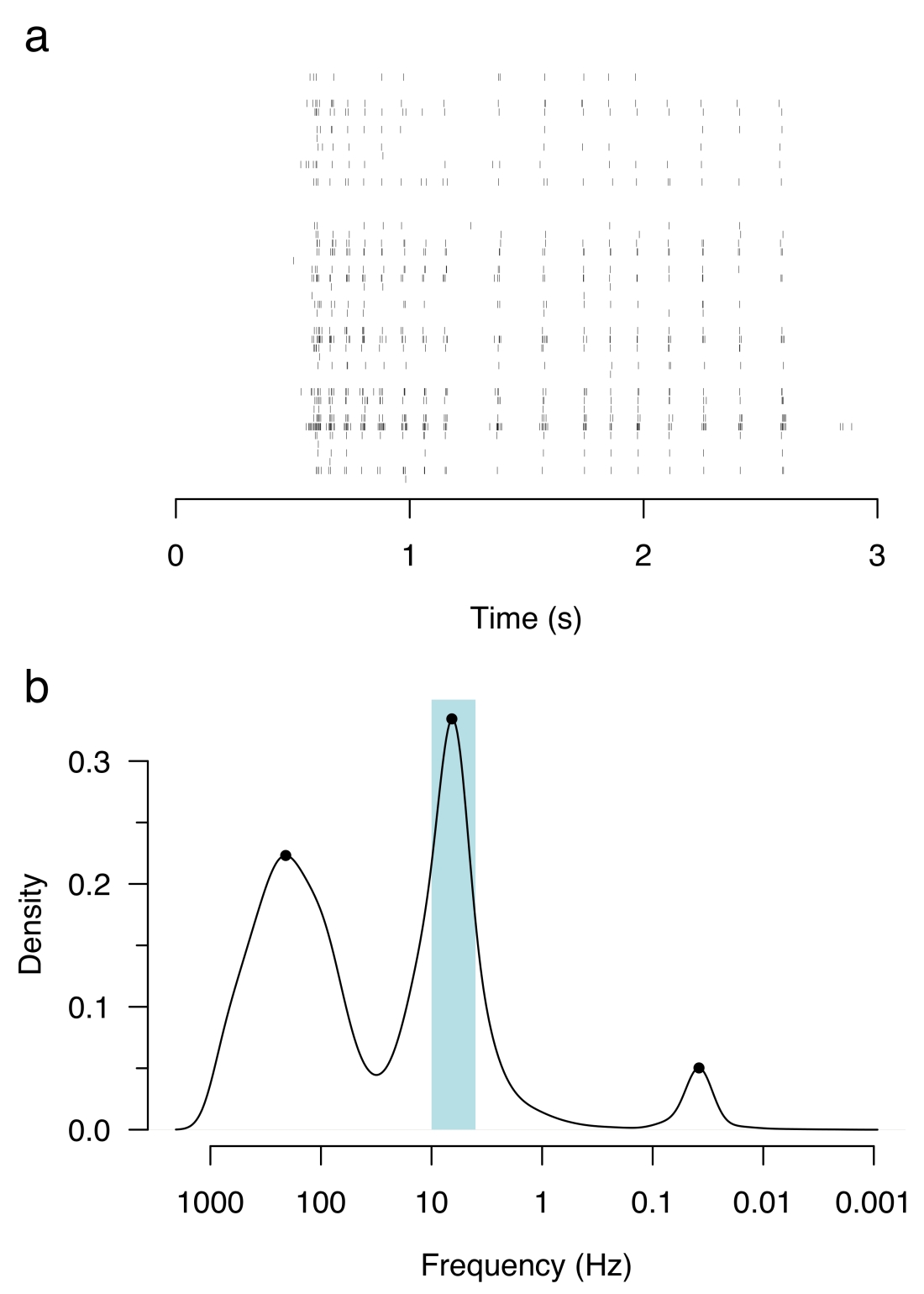
**

**Fig. S3.**

**Network spikes in wild-type and *Gria1*^-/-^ cultures**

**a.** Example of network spike analysis in wild type MEA-cultures (DIV 14). Detected network spikes are shown for 50s of recording, with the peak of each network spike denoted by a blue spot.

**b.** Detected network spikes in a *Gria1*^-/-^ network, shown for 50s of recording with the peak of each network spike denoted by a blue spot. Note how much activity in the *Gria1* mutant is sub-threshold and also that many suprathreshold activations only involve a subset of the available network.

**c.** Solid lines show mean of the total number of network spikes detected per 15 min recording epoch for *Gria1*^-/-^ cultures (red) vs wild-type cultures (black) plotted against culture age (DIV). Dashed lines show +/- 2.5% confidence interval of the bootstrapped mean.

**
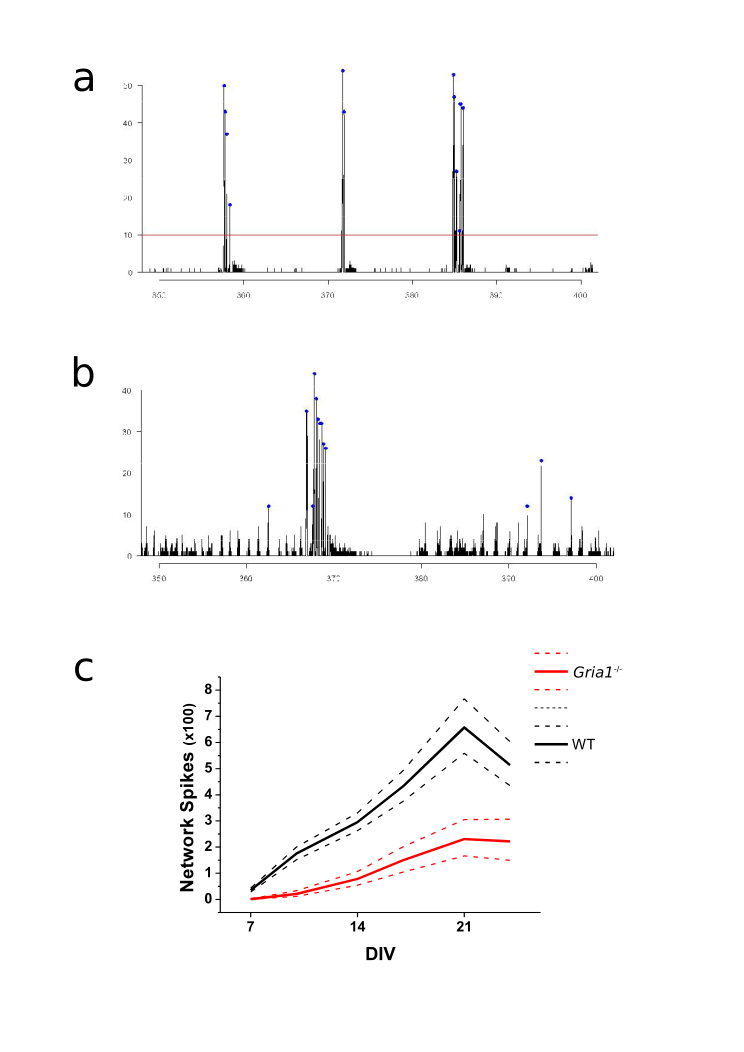
**

**Fig. S4.**

**Network activity is primarily mediated by glutamatergic neurotransmission.** Panels **a-d** show raster plots of 15 minute recordings, with the scale bar in panel **a** denoting 100s. Electrodes at which spontaneous activity was detected are referred to here as “active electrodes.” **a.** Control recording of a wild-type culture, aged 22 DIV (58 active electrodes) **b.** Bath application of DNQX (20μM) strongly reduced, but did not completely abolish, network activity (27 active electrodes). **c.** Co-application of DNQX (20μM) and APV (50μM) almost entirely eliminated activity in the network (3 active electrodes) **d.** Upon washout of the drugs, normal activity resumed (56 active electrodes).


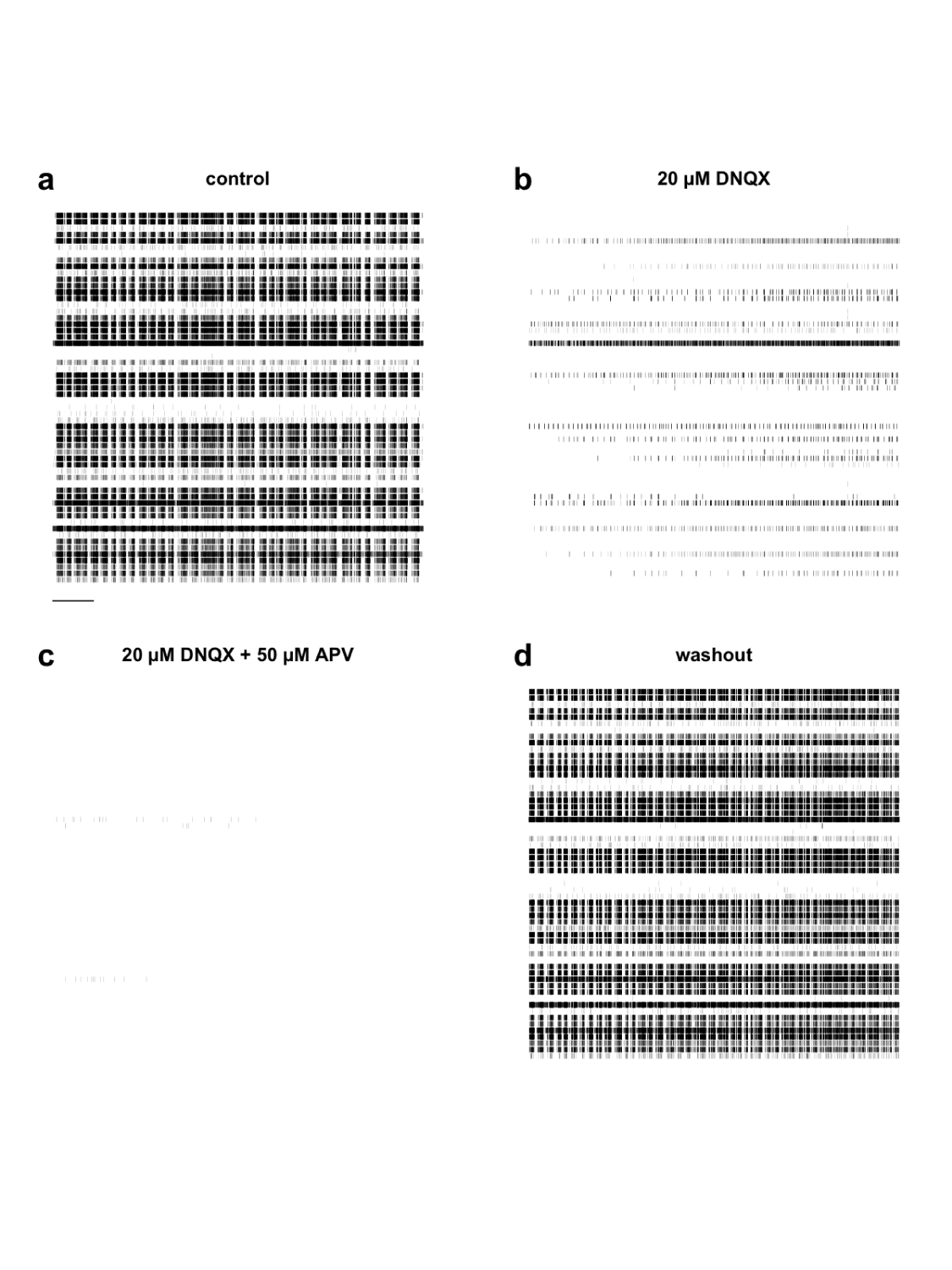
**Fig. S5.**

**Effect of varying threshold detection level on spike parameters. a-d**. Raster plots of 60 s of spikes recorded with detection threshold set to 0 µV in **a**, 20 µV **(**the standard default) in **b,** 30 µV in **c,** and 40 µV in **d**. Notice that while the total number of events detected reduces incrementally, the overall structure of the spike pattern remains relatively constant. Data from a single wild type (DIV 14) recording. Scale bar represents 10s.

**
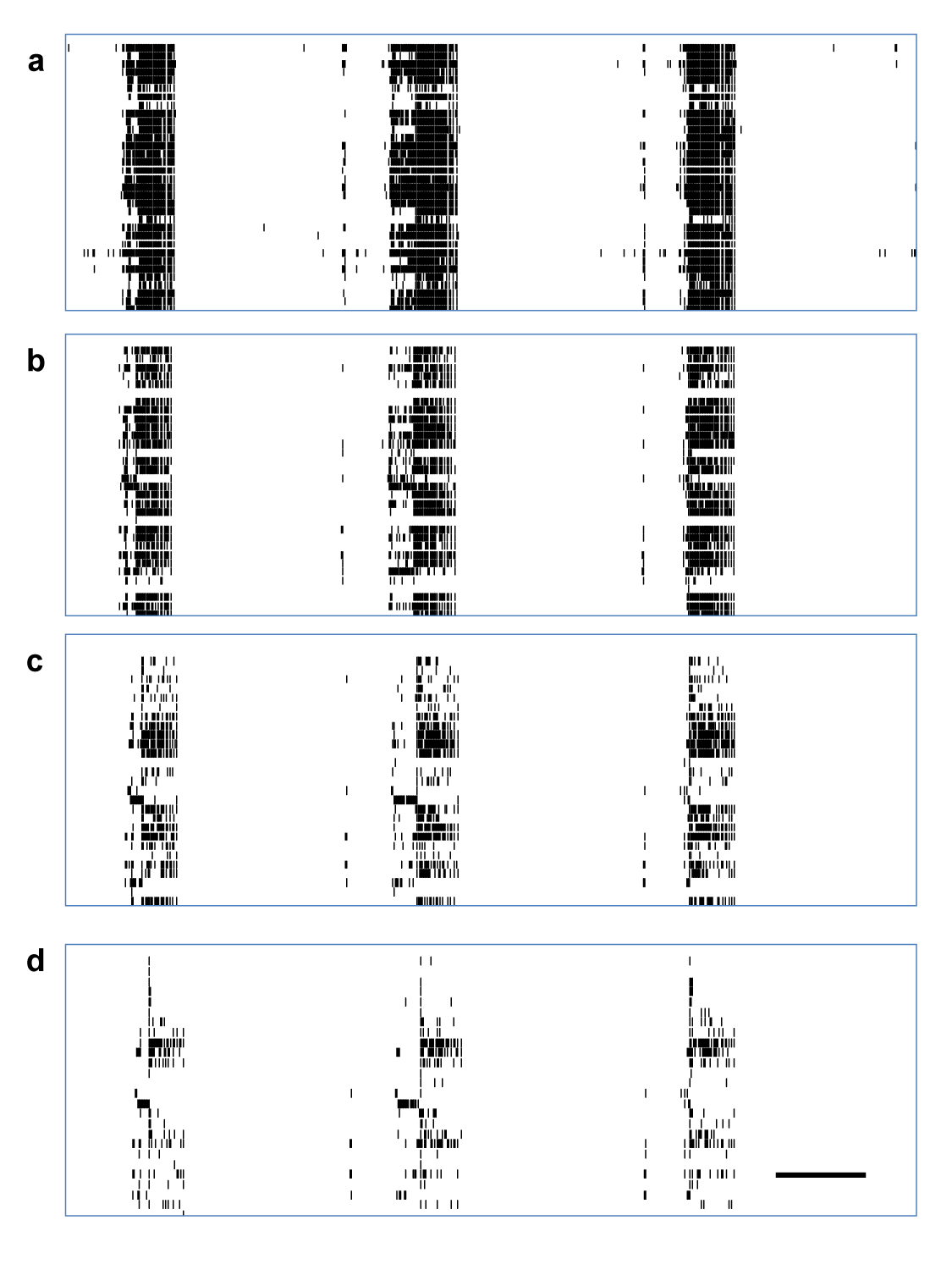
Fig. S6.**

**Developmental profiles of network activity in divergent wild type mouse strains. a.** Total spikes (sum of spikes recorded on all MEA channels throughout 15 min recording epoch). **b.** Network growth, assessed as the number of electrodes detecting > 1 burst per minute (maximum 59, the number of electrodes on an array). **c.** % spikes occurring within bursts. **d.** Burst rate (mean bursts per minute). In all graphs, the solid lines denote the mean value at each age and dashed lines denote 95% confidence intervals of the mean assessed by resampling.

**
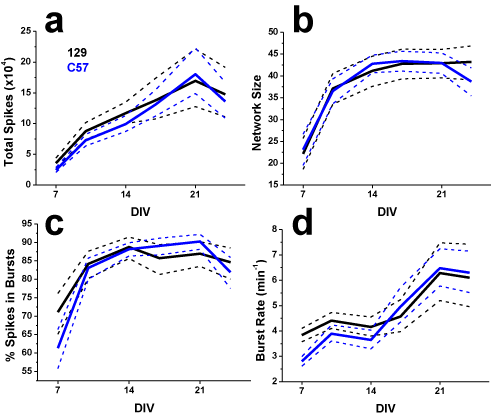
**

**Table S1. Summary of the mutant mouse lines used in the study.** Shown are the approved gene names for each mutant. Key: Layer (R = receptor; 1, 2, 3 = primary, secondary, tertiary interactor with NMDA receptor). Function GAP = GTPase activating protein (for rac / rap monomeric G protein) G beta, beta subunit of trimeric G protein. Phenotype shows total phenotype score, calculation detailed in Supplementary Methods and expanded in Supplementary Table 2.

| **Mutant** | **Gene** | **Layer** | **Function** | **Phenotype** | **Ref** | **#Cultures** | **#MEAs** |
| --- | --- | --- | --- | --- | --- | --- | --- |
| GLUA1 | *Gria1* | R | Receptor | 5.10 | [1] | 15 | 68 |
| APV | N/A | R | Receptor | 3.04 |  | 6 | 16 |
| \| PSD93 \|  \| \| --- \| --- \| | *Dlg2* | 1 | Scaffold | 1.69 | [2] | 10 | 71 |
| GRIT | *Arhgap32* | 1 | Rho GAP | 0.99 |  | 3 | 26 |
| PSD95 | *Dlg4* | 1 | Scaffold | 0.48 | [3] | 10 | 40 |
| SAP102 | *Dlg3* | 1 | Scaffold | 0.02 | [4] | 9 | 52 |
| SPAR | *Sipa1l1* | 2 | Rap GAP | 0.21 |  | 5 | 41 |
| GNB1 | *Gnb1* | 3 | G beta | 0.13 |  | 3 | 26 |

**References for Supplementary Table 1**

1. Zamanillo, D., et al., *Importance of AMPA receptors for hippocampal synaptic plasticity but not for spatial learning.* Science, 1999. **284**(5421): p. 1805-11.

2. McGee, A.W., et al., *PSD-93 knock-out mice reveal that neuronal MAGUKs are not required for development or function of parallel fiber synapses in cerebellum.* J Neurosci, 2001. **21**(9): p. 3085-91.

3. Migaud, M., et al., *Enhanced long-term potentiation and impaired learning in mice with mutant postsynaptic density-95 protein.* Nature, 1998. **396**(6710): p. 433-9.

4. Cuthbert, P.C., et al., *Synapse-associated protein 102/dlgh3 couples the NMDA receptor to specific plasticity pathways and learning strategies.* J Neurosci, 2007. **27**(10): p. 2673-82.

GRIT (*Arhgap32*), SPAR (Sipa1l1) and GNB1 (Gnb1) knockout mouse lines were generated within the Genes to Cognition programme at the Wellcome Trust Sanger Institute.

**Table S2. Mutant and APV network parameter datasets.** Complete data table for network parameters analyzed in all mutants and chronic APV-treated cultures. See Supplementary Methods for details of how calculations were made.

| ***Gria1*** | Active channels | | Total Spikes | | Network Size | | Burst Spikes | | Burst Pattern | | Corr. Index | | Burst Duration | | Burst Rate | | **Total** |
| --- | --- | --- | --- | --- | --- | --- | --- | --- | --- | --- | --- | --- | --- | --- | --- | --- | --- |
| DIV | Mut/WT | Score | Mut/WT | Score | Mut/WT | Score | Mut/WT | Score | Mut/WT | Score | Mut/WT | Score | Mut/WT | Score | Mut/WT | Score |  |
| 10 | 0.935 |  | 0.392 | 0.419 | 0.464 | 0.378 | 0.476 | 0.417 | 2.511 | 1.219 | 0.833 |  | 1.096 |  | 0.826 |  | 2.433 |
| 14 | 0.975 |  | 0.604 | 0.204 | 0.611 | 0.255 | 0.591 | 0.332 | 2.211 | 0.971 | 0.608 | 0.205 | 0.834 |  | 1.297 | 0.097 | 2.063 |
| 17 | 0.998 |  | 0.651 | 0.074 | 0.718 | 0.119 | 0.715 | 0.177 | 1.245 |  | 0.964 |  | 0.768 |  | 1.033 |  | 0.370 |
| 21 | 1.017 |  | 0.658 | 0.006 | 0.869 |  | 0.771 | 0.109 | 1.026 |  | 1.501 |  | 0.722 | 0.029 | 0.729 | 0.094 | 0.238 |
| 24 | 1.025 |  | 0.972 |  | 1.101 |  | 0.950 |  | 0.965 |  | 1.262 |  | 0.748 |  | 0.792 |  | 0.000 |
|  |  |  |  |  |  |  |  |  |  |  |  |  |  |  |  |  | **5.104** |
|  |  |  |  |  |  |  |  |  |  |  |  |  |  |  |  |  |  |
| **APV** | Active channels | | Total Spikes | | Network Size | | Burst Spikes | | Burst Pattern | | Corr. Index | | Burst Duration | | Burst Rate | | **Total** |
| DIV | Mut/WT | Score | Mut/WT | Score | Mut/WT | Score | Mut/WT | Score | Mut/WT | Score | Mut/WT | Score | Mut/WT | Score | Mut/WT | Score |  |
| 10 | 1.046 |  | 1.357 |  | 1.061 |  | 0.891 | 0.009 | 1.884 | 0.585 | 0.665 | 0.199 | 0.524 | 0.202 | 1.735 | 0.309 | 1.304 |
| 14 | 1.023 |  | 1.614 | 0.056 | 1.103 |  | 0.909 | 0.015 | 2.024 | 0.707 | 0.594 | 0.237 | 0.629 | 0.101 | 1.846 | 0.424 | 1.539 |
| 17 | 1.009 |  | 1.557 |  | 1.078 |  | 0.950 |  | 1.182 |  | 0.604 | 0.112 | 0.985 |  | 1.621 | 0.083 | 0.195 |
| 21 | 1.000 |  | 0.917 |  | 1.008 |  | 0.956 |  | 1.123 |  | 0.840 |  | 0.861 |  | 1.188 |  | 0.000 |
| 24 | 0.997 |  | 1.258 |  | 1.046 |  | 0.934 |  | 1.149 |  | 0.704 |  | 0.933 |  | 1.188 |  | 0.000 |
|  |  |  |  |  |  |  |  |  |  |  |  |  |  |  |  |  | **3.038** |
|  |  |  |  |  |  |  |  |  |  |  |  |  |  |  |  |  |  |
| ***Dlg2*** | Active channels | | Total Spikes | | Network Size | | Burst Spikes | | Burst Pattern | | Corr. Index | | Burst Duration | | Burst Rate | | **Total** |
| DIV | Mut/WT | Score | Mut/WT | Score | Mut/WT | Score | Mut/WT | Score | Mut/WT | Score | Mut/WT | Score | Mut/WT | Score | Mut/WT | Score |  |
| 10 | 0.933 |  | 0.701 | 0.070 | 0.820 | 0.003 | 0.861 | 0.057 | 1.437 | 0.298 | 0.743 | 0.146 | 0.773 | 0.028 | 1.082 |  | 0.602 |
| 14 | 0.958 |  | 0.998 |  | 0.894 |  | 0.915 | 0.021 | 1.346 | 0.097 | 0.680 | 0.198 | 1.203 |  | 1.176 |  | 0.316 |
| 17 | 0.964 |  | 1.348 |  | 0.923 |  | 0.959 |  | 1.246 |  | 0.490 | 0.347 | 1.471 | 0.115 | 1.327 | 0.037 | 0.500 |
| 21 | 0.948 |  | 0.968 |  | 0.877 |  | 0.891 | 0.032 | 1.217 |  | 0.616 | 0.152 | 1.210 |  | 1.114 |  | 0.184 |
| 24 | 0.953 |  | 0.947 |  | 0.829 |  | 0.875 | 0.010 | 1.366 | 0.069 | 0.733 | 0.013 | 1.295 |  | 0.971 |  | 0.092 |
|  |  |  |  |  |  |  |  |  |  |  |  |  |  |  |  |  | **1.693** |
|  |  |  |  |  |  |  |  |  |  |  |  |  |  |  |  |  |  |
| ***Arhgap32*** | Active channels | | Total Spikes | | Network Size | | Burst Spikes | | Burst Pattern | | Corr. Index | | Burst Duration | | Burst Rate | | **Total** |
| DIV | Mut/WT | Score | Mut/WT | Score | Mut/WT | Score | Mut/WT | Score | Mut/WT | Score | Mut/WT | Score | Mut/WT | Score | Mut/WT | Score |  |
| 10 | 0.967 |  | 0.790 |  | 0.903 |  | 0.902 | 0.013 | 1.288 | 0.106 | 0.859 |  | 0.807 |  | 1.090 |  | 0.119 |
| 14 | 0.981 |  | 0.909 |  | 0.921 |  | 0.905 | 0.025 | 1.289 | 0.022 | 0.962 |  | 0.659 | 0.170 | 1.296 | 0.034 | 0.251 |
| 17 | 0.993 |  | 0.877 |  | 0.952 |  | 0.949 |  | 1.129 |  | 0.889 |  | 0.971 |  | 1.085 |  | 0.000 |
| 21 | 0.999 |  | 1.012 |  | 0.928 |  | 0.966 |  | 1.759 | 0.220 | 0.556 | 0.160 | 1.617 | 0.039 | 1.080 |  | 0.418 |
| 24 | 1.007 |  | 1.078 |  | 1.004 |  | 1.029 |  | 1.501 | 0.100 | 0.752 |  | 1.680 | 0.100 | 1.035 |  | 0.200 |
|  |  |  |  |  |  |  |  |  |  |  |  |  |  |  |  |  | **0.988** |
|  |  |  |  |  |  |  |  |  |  |  |  |  |  |  |  |  |  |
| ***Dlg4*** | Active channels | | Total Spikes | | Network Size | | Burst Spikes | | Burst Pattern | | Corr. Index | | Burst Duration | | Burst Rate | | **Total** |
| DIV | Mut/WT | Score | Mut/WT | Score | Mut/WT | Score | Mut/WT | Score | Mut/WT | Score | Mut/WT | Score | Mut/WT | Score | Mut/WT | Score |  |
| 10 | 0.890 | 0.034 | 0.836 |  | 0.750 | 0.066 | 0.900 |  | 1.184 |  | 0.883 |  | 0.908 |  | 1.075 |  | 0.101 |
| 14 | 0.953 |  | 1.168 |  | 0.911 |  | 1.016 |  | 0.843 |  | 0.958 |  | 0.888 |  | 1.227 |  | 0.000 |
| 17 | 0.957 |  | 1.358 |  | 0.972 |  | 1.065 | 0.016 | 0.809 |  | 0.668 | 0.143 | 0.914 |  | 1.358 | 0.099 | 0.257 |
| 21 | 0.959 |  | 1.323 |  | 0.954 |  | 1.036 |  | 0.897 |  | 0.633 | 0.093 | 1.132 |  | 1.338 | 0.020 | 0.112 |
| 24 | 0.962 |  | 1.257 |  | 0.994 |  | 1.075 |  | 1.051 |  | 0.872 |  | 1.334 | 0.007 | 1.007 |  | 0.007 |
|  |  |  |  |  |  |  |  |  |  |  |  |  |  |  |  |  | **0.478** |
|  |  |  |  |  |  |  |  |  |  |  |  |  |  |  |  |  |  |
| ***Sipa1l1*** | Active channels | | Total Spikes | | Network Size | | Burst Spikes | | Burst Pattern | | Corr. Index | | Burst Duration | | Burst Rate | | **Total** |
| DIV | Mut/WT | Score | Mut/WT | Score | Mut/WT | Score | Mut/WT | Score | Mut/WT | Score | Mut/WT | Score | Mut/WT | Score | Mut/WT | Score |  |
| 10 | 0.916 |  | 0.859 |  | 0.859 |  | 0.920 | 0.013 | 1.208 | 0.019 | 0.853 |  | 1.138 |  | 1.044 |  | 0.032 |
| 14 | 0.908 | 0.020 | 0.990 |  | 0.814 | 0.030 | 0.924 | 0.013 | 1.346 | 0.095 | 0.877 |  | 1.203 |  | 1.085 |  | 0.159 |
| 17 | 0.921 | 0.013 | 0.782 |  | 0.889 |  | 0.956 |  | 0.884 |  | 1.181 |  | 0.782 |  | 0.946 |  | 0.013 |
| 21 | 0.938 | 0.005 | 0.946 |  | 0.939 |  | 0.980 |  | 1.236 |  | 1.123 |  | 1.065 |  | 0.939 |  | 0.005 |
| 24 | 0.953 |  | 1.508 |  | 1.012 |  | 1.056 |  | 1.369 |  | 0.874 |  | 1.232 |  | 1.151 |  | 0.000 |
|  |  |  |  |  |  |  |  |  |  |  |  |  |  |  |  |  | **0.209** |
|  |  |  |  |  |  |  |  |  |  |  |  |  |  |  |  |  |  |
| ***Gnb1*** | Active channels | | Total Spikes | | Network Size | | Burst Spikes | | Burst Pattern | | Corr. Index | | Burst Duration | | Burst Rate | | **Total** |
| DIV | Mut/WT | Score | Mut/WT | Score | Mut/WT | Score | Mut/WT | Score | Mut/WT | Score | Mut/WT | Score | Mut/WT | Score | Mut/WT | Score |  |
| 10 | 1.038 |  | 1.264 |  | 1.091 |  | 1.032 |  | 0.850 |  | 0.935 |  | 1.488 | 0.096 | 0.923 |  | 0.096 |
| 14 | 0.994 |  | 0.866 |  | 0.955 |  | 0.992 |  | 0.794 |  | 1.181 |  | 1.156 |  | 0.788 | 0.032 | 0.032 |
| 17 | 0.993 |  | 1.116 |  | 0.993 |  | 1.023 |  | 0.829 |  | 1.128 |  | 0.946 |  | 1.141 |  | 0.000 |
| 21 | 0.993 |  | 0.850 |  | 1.033 |  | 1.027 |  | 0.722 |  | 1.399 |  | 0.837 |  | 0.745 |  | 0.000 |
| 24 | 0.996 |  | 1.273 |  | 1.096 |  | 1.071 |  | 0.953 |  | 1.231 |  | 1.098 |  | 0.912 |  | 0.000 |
|  |  |  |  |  |  |  |  |  |  |  |  |  |  |  |  |  | **0.128** |
|  |  |  |  |  |  |  |  |  |  |  |  |  |  |  |  |  |  |
| ***Dlg3*** | Active channels | | Total Spikes | | Network Size | | Burst Spikes | | Burst Pattern | | Corr. Index | | Burst Duration | | Burst Rate | | **Total** |
| DIV | Mut/WT | Score | Mut/WT | Score | Mut/WT | Score | Mut/WT | Score | Mut/WT | Score | Mut/WT | Score | Mut/WT | Score | Mut/WT | Score |  |
| 10 | 1.023 |  | 1.157 |  | 1.077 |  | 0.969 |  | 1.062 |  | 0.939 |  | 0.897 |  | 1.106 |  | 0.000 |
| 14 | 1.003 |  | 1.230 |  | 1.029 |  | 0.953 |  | 1.158 |  | 0.919 |  | 0.891 |  | 1.209 | 0.018 | 0.018 |
| 17 | 1.003 |  | 1.354 |  | 1.063 |  | 0.989 |  | 1.110 |  | 0.939 |  | 1.093 |  | 1.038 |  | 0.000 |
| 21 | 0.993 |  | 0.973 |  | 1.041 |  | 0.970 |  | 0.882 |  | 1.332 | 0.002 | 0.775 |  | 0.886 |  | 0.002 |
| 24 | 1.004 |  | 1.138 |  | 1.113 |  | 1.045 |  | 1.018 |  | 1.210 |  | 1.123 |  | 0.953 |  | 0.000 |
|  |  |  |  |  |  |  |  |  |  |  |  |  |  |  |  |  | **0.020** |
